# The C-reactive protein–albumin–lymphocyte index is associated with ventricular functional improvement and long-term outcomes after cardiac resynchronization therapy

**DOI:** 10.3389/fnut.2026.1961200

**Published:** 2026-09-18

**Authors:** Run Zhang, Qingqing Duan, Xin Lv, Yuchen Liu, Weirui Chen, Zhihui Yan, Jia Liu, Shuaishuai Wang, Dan Zhao, Qing Zhao, Wenzhong Zhang

**Affiliations:** 1 Department of Cardiology, The Affiliated Hospital of Qingdao University, Qingdao, China; 2 Department of Cardiology, Qingdao Cardiovascular Hospital, Qingdao, China; 3 Department of Cardiology, Linzi District Maternal and Child Health Hospital (Qidu Hospital), Zibo, China; 4 Department of Cardiology, Laizhou People’s Hospital, Yantai, Shandong, China

**Keywords:** cardiac resynchronization therapy, C-reactive protein–albumin–lymphocyte index, heart failure, inflammation, nutritional status, prognosis

## Abstract

**Background and aims:**

Inflammation, malnutrition, and immune dysfunction are closely linked to adverse outcomes in heart failure. The C-reactive protein–albumin–lymphocyte (CALLY) index integrates these biological domains, but its clinical relevance in patients undergoing cardiac resynchronization therapy (CRT) remains insufficiently characterized. We investigated whether CALLY is associated with left ventricular ejection fraction (LVEF) improvement and long-term clinical outcomes after CRT.

**Methods:**

In this multicenter retrospective cohort study, 213 consecutive patients who underwent CRT implantation between January 2015 and August 2025 were enrolled. The CALLY index was analyzed primarily as a log2-transformed continuous variable. The primary endpoint was time to first major adverse cardiovascular event (MACE), defined as all-cause mortality, heart transplantation, left ventricular assist device implantation, or heart-failure rehospitalization. Echocardiographic response was evaluated in 191 patients with follow-up imaging. Multivariable Cox and linear regression were used to assess associations with MACE and absolute change in LVEF, respectively. Sensitivity analyses addressed baseline LVEF, pacemaker upgrade status, calendar era, and alternative handling of CR*p* values at the lower detection limit.

**Results:**

During a median follow-up of 18 months, 56 patients (26.3%) experienced MACE, including 48 heart-failure rehospitalizations and 8 deaths. Each doubling of baseline CALLY was associated with a lower hazard of MACE (hazard ratio, 0.751; 95% confidence interval, 0.664–0.850; *p* < 0.001) and greater LVEF improvement (*β* = 0.949 percentage points; 95% confidence interval, 0.313–1.586; *p* = 0.004). Addition of CALLY to a parsimonious clinical model increased the Harrell C-index from 0.672 to 0.738; the optimism-corrected C-index was 0.705. Time-dependent AUCs were 0.773 and 0.759 at 12 and 18 months, respectively. The findings remained consistent after restriction to patients with LVEF ≤35%, exclusion of pacemaker-upgrade patients, adjustment or stratification by calendar era, and alternative approaches to CR*p* values at the lower detection limit, with no significant interactions by baseline LVEF or calendar era.

**Conclusion:**

A higher baseline CALLY index was independently associated with greater LVEF improvement and a lower hazard of adverse cardiovascular outcomes after CRT. CALLY may provide complementary prognostic information in patients undergoing CRT, although prospective external validation is required before clinical implementation.

## 1 Introduction

Heart failure (HF) remains a leading cause of morbidity, mortality, and healthcare utilization worldwide despite substantial advances in pharmacological and device-based therapies (1–3). Cardiac resynchronization therapy (CRT) is an established treatment for patients with heart failure with reduced ejection fraction and electrical dyssynchrony, improving left ventricular reverse remodeling, functional status, and long-term survival (4, 5). Despite these benefits, approximately one-third of patients fail to achieve a meaningful clinical or echocardiographic response after CRT implantation (6, 7). Current approaches to patient selection and response prediction rely primarily on electrocardiographic and echocardiographic characteristics, such as QRS duration, left bundle branch block morphology, and left ventricular function (4, 5, 7). However, these parameters mainly reflect cardiac electrical and mechanical abnormalities and provide limited insight into the systemic biological status of patients, which may contribute to the substantial heterogeneity in CRT response.

Chronic inflammation, malnutrition, and immune dysfunction are increasingly recognized as interconnected processes that play central roles in the progression of HF and its clinical outcomes (8–11). Persistent inflammatory activation promotes myocardial fibrosis, adverse ventricular remodeling, and neurohormonal activation, whereas impaired nutritional status and immune competence reduce physiological reserve and may limit myocardial recovery. Because these biological processes frequently coexist and interact, biomarkers integrating inflammation, nutritional status, and immune function may provide more comprehensive prognostic information than individual biomarkers alone.

The C-reactive protein–albumin–lymphocyte (CALLY) index is a composite biomarker calculated from routinely available laboratory parameters that reflects the combined effects of inflammation, nutritional status, and immune competence. Previous studies have reported associations between the CALLY index and clinical outcomes in patients with heart failure, acute coronary syndrome, atrial fibrillation, chronic obstructive pulmonary disease, and several malignancies (12–16). More recently, a large cohort study of patients with chronic heart failure demonstrated an independent association between higher CALLY levels and more favorable cardiovascular outcomes (17). Compared with isolated inflammatory or nutritional markers, the CALLY index may better capture the overall systemic biological condition of patients. Nutritional indices such as the geriatric nutritional risk index (GNRI) have also been associated with treatment response and long-term prognosis after CRT (18). Similarly, the Controlling Nutritional Status (CONUT) score has been associated with CRT response and long-term outcomes, further supporting the prognostic relevance of nutritional status in this population (19). However, the association between the CALLY index and left ventricular ejection fraction (LVEF)-based echocardiographic response and clinical outcomes after CRT has not been well characterized.

In this multicenter retrospective cohort study, we investigated the association of the baseline CALLY index with LVEF-based echocardiographic response and long-term clinical outcomes after CRT implantation. We hypothesized that a higher baseline CALLY index, reflecting a more favorable inflammation–nutrition–immune profile, would be associated with greater improvement in LVEF and a lower hazard of major adverse cardiovascular events following CRT.

## 2 Materials and methods

### 2.1 Study design and population

This multicenter retrospective cohort study included consecutive patients who underwent cardiac resynchronization therapy (CRT) at three tertiary hospitals in Qingdao, China, between January 1, 2015, and August 1, 2025. CRT implantation was performed according to guideline recommendations and clinical indications applicable at the time. Because the study spanned 2015–2025, the 2021 European Society of Cardiology (ESC) criteria were not retrospectively applied to the entire cohort. The study therefore reflected real-world CRT practice, including conventional heart-failure indications and pacing-related indications such as upgrade from a pre-existing permanent pacemaker. Fourteen patients underwent upgrade from a pre-existing permanent pacemaker to CRT.

Among the 239 patients initially screened, 10 were excluded because of extensive missing baseline information that precluded reliable clinical characterization and inclusion in the planned analyses. Other exclusions included advanced malignancy, autoimmune disease, end-stage liver disease, end-stage kidney disease, active infection or clinically evident acute inflammatory conditions at baseline blood sampling, and loss to follow-up. The remaining 213 patients were included in the final analysis (Figure 1). Of these, 171, 25, and 17 patients were enrolled from the three participating centers. Baseline demographic, clinical, laboratory, electrocardiographic, and echocardiographic data required for the main analyses were complete in the final cohort. Follow-up echocardiographic data were unavailable in 22 patients, as described below.

**Figure 1:**
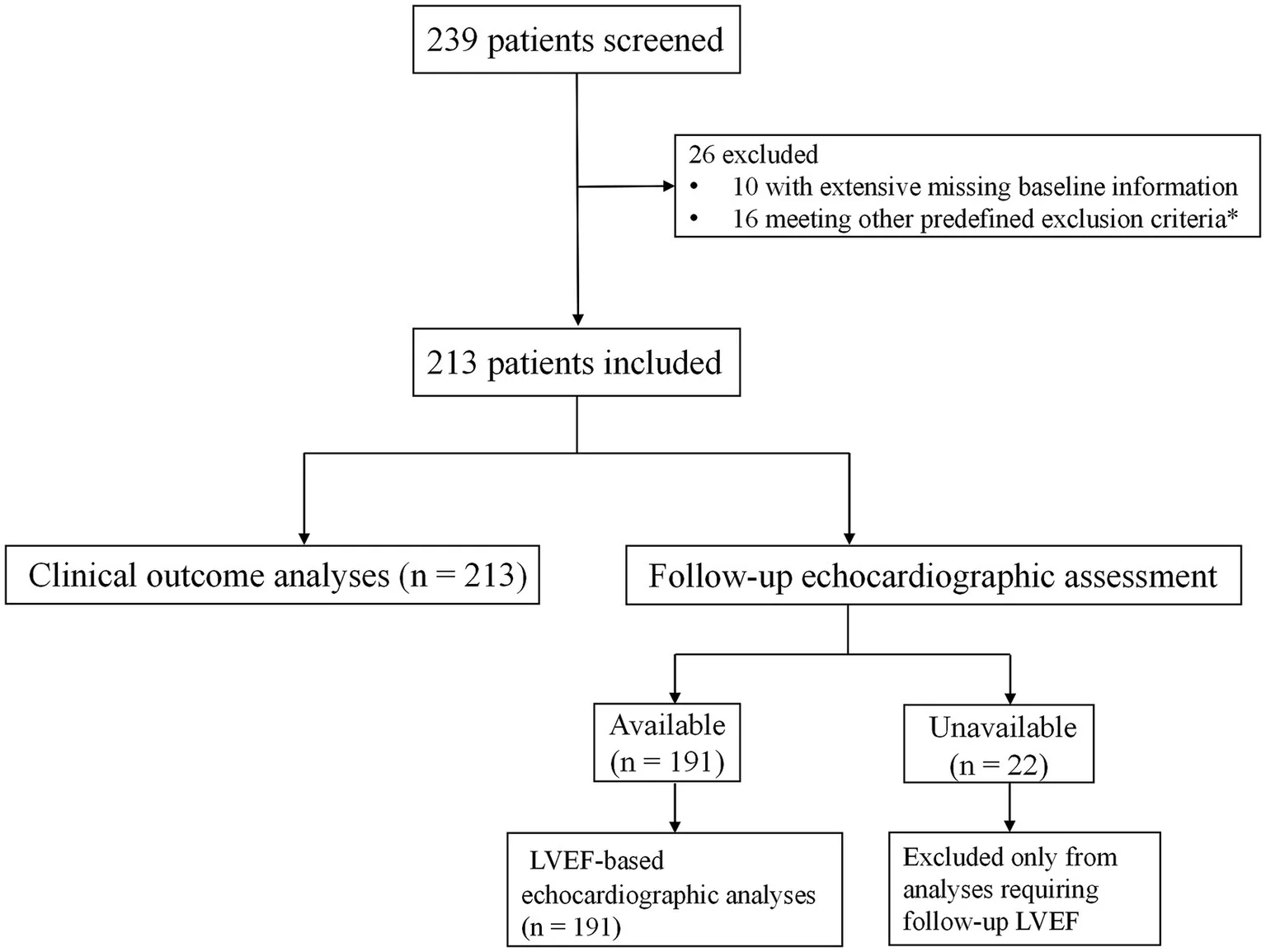
Study flowchart of patient selection and analysis cohorts. Among 239 patients screened, 26 were excluded, including 10 with extensive missing baseline information that precluded inclusion in the planned analyses and 16 meeting other predefined exclusion criteria. The remaining 213 patients were included in the clinical outcome analyses. Follow-up echocardiographic data were available in 191 patients, who were included in the LVEF-based echocardiographic analyses; 22 patients without follow-up echocardiography were excluded only from analyses requiring follow-up LVEF. *Other predefined exclusion criteria included advanced malignancy, autoimmune disease, end-stage liver disease, end-stage kidney disease, active infection or clinically evident acute inflammatory conditions at baseline blood sampling, and loss to follow-up. LVEF, left ventricular ejection fraction.

The study was conducted in accordance with the Declaration of Helsinki and was approved by the Ethics Committee of the Affiliated Hospital of Qingdao University. The requirement for written informed consent was waived owing to the retrospective nature of the study and the use of de-identified data.

### 2.2 Clinical data collection and calculation of the CALLY index

Baseline demographic characteristics, medical history, cardiovascular risk factors, medication use, laboratory measurements, electrocardiographic findings, and echocardiographic parameters were retrieved from the electronic medical record systems of the participating hospitals by trained investigators.

Arrhythmia was recorded as a binary variable indicating documented rhythm disorders, whereas a history of atrial fibrillation (AF) was identified separately from baseline medical records.

Fasting peripheral venous blood samples were collected 1 day before CRT implantation. Standard C-reactive protein (CRP), rather than high-sensitivity CRP, was measured. Serum albumin and complete blood cell counts, including lymphocyte counts, were also measured according to standardized laboratory procedures at each participating center. The lower detection limit for CRP was 0.5 mg/L. Values at or below the lower detection limit were assigned 0.5 mg/L for calculation of the CALLY index. Patients with active infection or clinically evident acute inflammatory conditions at the time of blood sampling were excluded.

The C-reactive protein–albumin–lymphocyte (CALLY) index was calculated as follows:

CALLY index = [serum albumin (g/L) × lymphocyte count (×10^9^/L)]/[CRP (mg/L) × 10].

Because the CALLY index was right-skewed, it was log2-transformed for continuous analyses. Effect estimates for log2-transformed CALLY therefore represent the change in outcome associated with each doubling of the CALLY index.

For comparison, four additional inflammatory and nutritional indices were calculated: the inflammatory burden index (IBI), platelet-to-lymphocyte ratio (PLR), prognostic nutritional index (PNI), and geriatric nutritional risk index (GNRI). Body mass index (BMI) was used in the calculation of GNRI. The indices were calculated as follows:

IBI = CRP (mg/L) × [neutrophil count (×10^9^/L)/lymphocyte count (×10^9^/L)];

PLR = platelet count (×10^9^/L) / lymphocyte count (×10^9^/L);

PNI = serum albumin (g/L) + 5 × lymphocyte count (×10^9^/L);

GNRI = 1.489 × serum albumin (g/L) + 41.7 × min [1, BMI / 22].

### 2.3 Study endpoints and follow-up

Patients were followed through scheduled outpatient visits or structured telephone interviews conducted by trained physicians. The primary endpoint was time to the first major adverse cardiovascular event (MACE), defined as a composite of all-cause mortality, heart transplantation, left ventricular assist device implantation, or heart-failure rehospitalization during follow-up. For time-to-event analyses, only the first occurrence of any component of the composite endpoint was considered.

Heart-failure rehospitalization was defined as an unplanned hospital admission lasting at least 24 h (or extending across a calendar date) for new or worsening signs and/or symptoms of heart failure requiring initiation or intensification of heart-failure–directed therapy. Heart-failure rehospitalization and all-cause death were also recorded separately for analyses of individual event components and competing risks.

The secondary endpoint was LVEF-based echocardiographic response to CRT. Echocardiographic examinations performed 6–12 months after CRT implantation were reviewed when available. The principal echocardiographic outcome was the absolute change in LVEF (ΔLVEF), calculated as follow-up LVEF minus baseline LVEF. For descriptive categorization of LVEF-based response, super-response was defined as an absolute increase in LVEF ≥15%; response as an increase of ≥5% but <15%; and non-response as an increase <5%. Follow-up echocardiographic data were available for 191 patients; the remaining 22 patients without follow-up echocardiographic measurements were excluded from analyses requiring follow-up LVEF.

All clinical events and echocardiographic outcomes were independently adjudicated by two cardiologists. Disagreements were resolved by consensus with a senior cardiologist.

### 2.4 Statistical analysis

Statistical analyses were performed using SPSS version 29.0 (IBM Corp., Armonk, NY, United States) and R version 4.5.2 (R Foundation for Statistical Computing, Vienna, Austria). All tests were two-sided, and a *p* < 0.05 was considered statistically significant. Continuous variables were assessed for normality using the Shapiro–Wilk test and are presented as the mean ± standard deviation (SD) or median (interquartile range [IQR]), as appropriate. Comparisons between patients with and without MACE were performed using Student’s *t*-test or the Mann–Whitney U test. Categorical variables are presented as frequencies and percentages and were compared using the chi-square test or Fisher’s exact test, as appropriate. Baseline characteristics were also compared between patients with and without follow-up echocardiographic data using Student’s *t-*test or the Mann–Whitney U test for continuous variables and the chi-square test or Fisher’s exact test for categorical variables, as appropriate. Baseline variables required for the main analyses were complete in the final cohort. Follow-up echocardiographic data were unavailable in 22 patients; these patients were excluded only from analyses requiring follow-up LVEF, and no imputation was performed.

Because the CALLY index was right-skewed, it was log2-transformed for continuous analyses, such that effect estimates represent the change associated with each doubling of CALLY. N-terminal pro-B-type natriuretic peptide (NT-proBNP) was also log2-transformed because of its skewed distribution. CALLY tertiles were retained for descriptive comparisons and Kaplan–Meier analyses. Tertile boundaries were determined from the baseline CALLY distribution in the full cohort of 213 patients, with cutoffs of 2.41 and 8.92.

Time-to-event outcomes were analyzed using the Kaplan–Meier method and compared with the log-rank test. The independent association between the CALLY index and MACE was evaluated using Cox proportional hazards regression models. Model 0 included log2-transformed CALLY alone; Model 1 additionally included age and sex; and the principal Model 2 further included ischemic versus nonischemic etiology, left bundle branch block (LBBB), baseline LVEF, and log2-transformed NT-proBNP. Individual components of the CALLY index were not entered simultaneously with CALLY. The proportional hazards assumption was assessed using Schoenfeld residuals.

To assess the incremental prognostic information provided by CALLY, a parsimonious clinical Cox model including age, sex, ischemic versus nonischemic etiology, left bundle branch block, baseline LVEF, and log2-transformed NT-proBNP was compared with the same model additionally including log2-transformed CALLY. The change in Harrell’s C-index and its 95% confidence interval (CI) were estimated using 1,000 bootstrap resamples, and the models were compared using the likelihood-ratio test. The principal model was also internally validated using 1,000 bootstrap resamples to estimate optimism and obtain an optimism-corrected Harrell C-index.

Because heart-failure rehospitalization accounted for the majority of MACE events and death could preclude subsequent rehospitalization, a Fine–Gray competing-risk model was used to evaluate first heart-failure rehospitalization, with all-cause death treated as a competing event. The competing-risk model used the same covariates as the principal Cox model.

For the echocardiographic analysis, absolute change in LVEF (follow-up LVEF minus baseline LVEF) was analyzed as a continuous outcome. Its association with log2-transformed CALLY was evaluated using unadjusted and multivariable linear regression. The adjusted model included age, sex, ischemic versus nonischemic etiology, left bundle branch block, baseline LVEF, and preprocedural QRS duration. Heteroskedasticity-consistent (HC3) robust standard errors were used, and variance inflation factors were examined to assess multicollinearity.

Time-dependent receiver operating characteristic analyses were performed at 12 and 18 months to compare the discrimination of the CALLY index with the inflammatory burden index (IBI), platelet-to-lymphocyte ratio (PLR), geriatric nutritional risk index (GNRI), and prognostic nutritional index (PNI) for MACE. Pairwise comparisons between CALLY and the other biomarkers were adjusted for multiple testing using the Holm method.

Restricted cubic spline analysis was performed to characterize the shape of the association between the CALLY index and MACE. The spline model used log2-transformed CALLY with three knots placed at the 10th, 50th, and 90th percentiles and was adjusted for the same covariates as the principal Cox model. Overall and nonlinear associations were tested separately.

Exploratory subgroup analyses were conducted according to age, sex, body mass index, hypertension, diabetes mellitus, coronary heart disease, and arrhythmia status. CALLY was analyzed continuously on the log2 scale in subgroup analyses, and effect estimates therefore represent the hazard ratio per doubling of CALLY. Potential effect modification was evaluated using interaction terms and likelihood-ratio tests.

Additional sensitivity analyses were performed to assess the potential influence of heterogeneity in CRT indications and calendar era. The principal Cox and echocardiographic analyses were repeated after restriction to patients with baseline LVEF ≤35% and after exclusion of patients upgraded from a pre-existing pacemaker. Analyses were also stratified by baseline LVEF (≤35% vs. >35%), with formal interaction testing. To assess temporal effects, the principal models were additionally adjusted for calendar era (2015–2020 vs. 2021–2025) or implantation year, and Cox analyses were further adjusted for baseline SGLT2 inhibitor use. Stratified analyses and interaction tests were also performed according to calendar era.

To assess the influence of CR*p* values at the lower detection limit, sensitivity analyses replaced these values with LOD/√2 or LOD/2, followed by recalculation of CALLY and repetition of the principal Cox and echocardiographic analyses. An additional sensitivity analysis used left-censored lognormal multiple imputation with 100 imputations.

## 3 Results

### 3.1 Baseline characteristics

The baseline characteristics of the study population are summarized in Table 1. Among the 213 patients included in the analysis, 56 (26.3%) experienced MACE during follow-up, whereas 157 (73.7%) remained event-free.

**Table 1:** Baseline characteristics of patients according to the occurrence of major adverse cardiovascular events (MACE).

Variables	All (*n* = 213)	MACE (*n* = 56)	No MACE (*n* = 157)	*P*-value
Age, years	63(57, 69)	63(56, 71)	63(57, 69)	0.920
Male sex	149(70.0)	46(82.1)	103(65.6)	**0.032**
BMI, kg/m^2^	24.2(22.0, 26.5)	25.2(22.8, 27.5)	24.1(22.0, 26.3)	0.096
Smoking	72(33.8)	23(41.1)	49(31.2)	**0.024**
Drinking	38(17.8)	12(21.4)	26(16.6)	0.539
Hypertension	97(45.5)	27(48.2)	70(44.6)	0.755
DM	48(22.5)	12(21.4)	36(23.0)	0.964
CHD	104(48.8)	26(46.4)	78(50.0)	0.793
Arrhythmia	180(84.5)	50(89.3)	130(82.8)	0.349
AF	41(19.2)	12(21.4)	29(18.5)	0.630
COPD	3(1.4)	2(3.6)	1(0.6)	0.347
Renal insufficiency	15(7.0)	7(12.5)	8(5.1)	0.120
**CRT device type**				0.052
CRT-D	123(57.7)	39(69.6)	84(53.5)	
CRT-P	90(42.3)	17(30.4)	73(46.5)	
**NYHA class**				**0.006**
II	47(22.1)	6(10.7)	41(26.1)	
III	121(56.8)	33(58.9)	88(56.1)	
IV	45(21.1)	17(30.4)	28(17.8)	
**Cardiac parameters**				
QRSd, ms	164.0(152.0, 177.0)	160.0(150.0, 172.8)	166.0(152.0, 181.5)	0.059
LBBB	121(56.8)	37(66.1)	84(53.5)	0.141
LVEF, %	31.0(25.5, 38.0)	30.00(25.0, 37.8)	31.0(26.0, 38.0)	0.501
LVEDD, mm	63.0(57.0, 72.0)	65.00(60.0, 75.8)	62.0(57.0, 71.5)	0.130
LAD, mm	55.0(46.0, 62.0)	55.00(46.3, 62.0)	55.0(46.0, 62.0)	0.815
**Laboratory tests**				
WBC	6.33(5.27, 7.59)	6.25(5.32, 7.53)	6.37(5.24, 7.65)	0.953
Lymphocyte	1.71(1.34, 2.07)	1.52(1.23, 1.90)	1.74(1.43, 2.11)	**0.013**
Neutrophils	3.94(2.99, 5.02)	4.05(3.27, 5.17)	3.85(2.86, 5.00)	0.295
Hb	139.0(127.0, 150.0)	132.5(126.0, 147.0)	139.0(128.5, 150.0)	0.183
Platelets	194.0(155.5, 235.5)	181.5(147.0, 234.3)	199.0(163.5, 236.0)	0.352
Albumin	39.61 ± 4.32	38.49 ± 4.04	40.01 ± 4.36	**0.024**
Creatinine, μmol/L	80.0(67.5, 98.6)	94.0(76.6, 111.9)	76.8(62.5, 91.9)	**<0.001**
TC, mmol/L	3.95(3.32, 4.75)	3.55(3.14, 4.72)	4.08(3.35, 4.75)	0.184
TG, mmol/L	1.14(0.80, 1.51)	1.17(0.82, 1.49)	1.14(0.79, 1.52)	0.726
HDL-C, mmol/L	1.11(0.93, 1.32)	1.02(0.88, 1.18)	1.14(0.98, 1.39)	**0.005**
LDL-C, mmol/L	2.26(1.74, 2.94)	2.05(1.71, 2.98)	2.31(1.75, 2.94)	0.630
NT-proBNP, pg./mL	923.0(356.1, 1893.4)	1444.5(489.1, 2605.3)	827.0(323.7, 1728.5)	**0.012**
**Medications**				
RAAS inhibitor	149(70.0)	35(62.5)	114(72.6)	0.212
Beta-blocker	168(78.9)	44(78.6)	124(79.0)	0.949
Spironolactone	185(86.9)	50(89.3)	135(86.0)	0.691
SGLT-2i	86(40.4)	18(32.1)	68(43.3)	0.192
**Inflammatory and nutritional indices**				
CALLY	4.66 (1.50, 11.85)	1.72(0.84, 3.73)	6.83(2.80, 12.86)	**<0.001**
IBI	3.26(1.03, 10.35)	8.89(3.73, 27.09)	2.33(0.93, 6.39)	**<0.001**
PLR	109.42(91.01, 147.27)	124.71(89.22, 172.09)	108.97(91.93, 139.60)	0.208
GNRI	100.22 (95.12, 103.94)	99.34 (94.89, 102.78)	100.74 (95.45, 104.39)	0.053
PNI	48.45 (44.89, 52.01)	46.33 (43.99, 49.88)	49.45 (45.29, 52.98)	**0.004**

Data are presented as mean ± SD, median (interquartile range), or n (%), as appropriate. MACE, major adverse cardiovascular events; BMI, body mass index; CRT, cardiac resynchronization therapy; CRT-D, cardiac resynchronization therapy defibrillator; CRT-P, cardiac resynchronization therapy pacemaker; DM, diabetes mellitus; CHD, coronary heart disease; AF, atrial fibrillation; COPD, chronic obstructive pulmonary disease; NYHA, New York Heart Association; QRSd, QRS duration; LBBB, left bundle branch block; LVEF, left ventricular ejection fraction; LVEDD, left ventricular end-diastolic diameter; LAD, left atrial diameter; WBC, white blood cell count; Hb, hemoglobin; TC, total cholesterol; TG, triglyceride; HDL-C, high-density lipoprotein cholesterol; LDL-C, low-density lipoprotein cholesterol; NT-proBNP, N-terminal pro-B-type natriuretic peptide; RAAS, renin–angiotensin–aldosterone system; SGLT-2i, sodium–glucose cotransporter 2 inhibitor; CALLY, C-reactive protein–albumin–lymphocyte index; IBI, inflammatory burden index; PLR, platelet-to-lymphocyte ratio; GNRI, geriatric nutritional risk index; PNI, prognostic nutritional index. Bold indicates *P* < 0.05.

Compared with patients without MACE, those who experienced MACE were more frequently male and current smokers and had more advanced HF, as indicated by a higher proportion of New York Heart Association (NYHA) class IV. Atrial fibrillation was present in 41 patients (19.2%) at baseline, with no significant difference between the MACE and non-MACE groups (21.4% vs. 18.5%, *p* = 0.630). Laboratory findings showed significantly lower lymphocyte counts, serum albumin, and high-density lipoprotein cholesterol (HDL-C) levels, together with higher serum creatinine and N-terminal pro-B-type natriuretic peptide (NT-proBNP) concentrations in the MACE group.

Among the inflammatory and nutritional biomarkers evaluated, the CALLY index, inflammatory burden index (IBI), and prognostic nutritional index (PNI) differed significantly between groups, whereas the geriatric nutritional risk index (GNRI) and platelet-to-lymphocyte ratio (PLR) did not. Patients who developed MACE had significantly lower baseline CALLY and PNI values and higher IBI values than those who remained event-free. These findings indicate that differences in baseline inflammation–nutrition-related biomarker profiles were associated with subsequent clinical outcomes.

### 3.2 Association of the CALLY index with LVEF-based echocardiographic response

Follow-up echocardiographic data were available for 191 of the 213 patients. In these patients, a higher baseline CALLY index was associated with greater improvement in LVEF (Figure 2). In the unadjusted linear regression analysis, each doubling of the CALLY index was associated with a 0.90-percentage-point greater increase in LVEF (*β* = 0.897; 95% CI, 0.291–1.503; *p* = 0.004).

**Figure 2:**
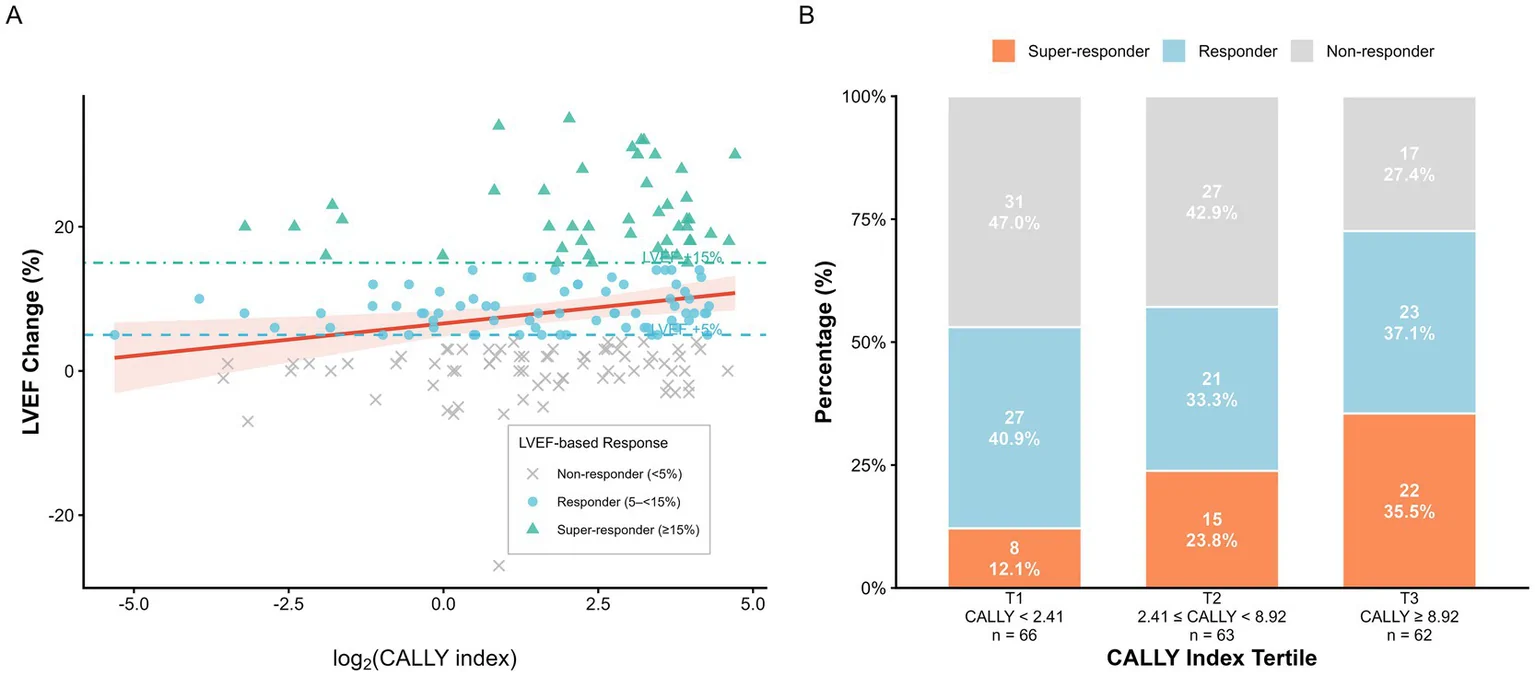
Association between baseline CALLY index and LVEF-based echocardiographic response after cardiac resynchronization therapy. **(A)** Relationship between log2-transformed baseline CALLY index and absolute change in LVEF at 6–12 months after CRT among the 191 patients with follow-up echocardiography. The solid line and shaded area represent the fitted linear regression line and 95% confidence interval, respectively. **(B)** Distribution of LVEF-based response categories across baseline CALLY tertiles in the 191 patients with follow-up echocardiography. CALLY tertile cutoffs (2.41 and 8.92) were derived from the baseline CALLY distribution in the full cohort of 213 patients and then applied to the echocardiographic analysis cohort, resulting in 66, 63, and 62 patients in the lowest, middle, and highest tertiles, respectively. CALLY, C-reactive protein–albumin–lymphocyte index; CRT, cardiac resynchronization therapy; LVEF, left ventricular ejection fraction.

After adjustment for age, sex, ischemic etiology, left bundle branch block, baseline LVEF, and preprocedural QRS duration, the association remained significant. Each doubling of the baseline CALLY index was associated with a 0.95-percentage-point greater increase in LVEF (*β* = 0.949; 95% CI, 0.313–1.586; *p* = 0.004). Baseline LVEF was inversely associated with subsequent LVEF improvement (*β* = −0.293; 95% CI, −0.477 to −0.109; *p* = 0.002). No substantial multicollinearity was observed among model covariates (all variance inflation factors <1.21) (Table 2).

**Table 2:** Association between baseline CALLY index and improvement in LVEF.

Model	β per doubling of CALLY	95% CI	*P*-value
Unadjusted	0.897	0.291–1.503	0.004
Adjusted	0.949	0.313–1.586	0.004

The adjusted model included age, sex, ischemic versus nonischemic etiology, left bundle branch block, baseline LVEF, and preprocedural QRS duration. CALLY was analyzed after log2 transformation; therefore, β represents the absolute difference in LVEF improvement associated with each doubling of the CALLY index. CALLY, C-reactive protein–albumin–lymphocyte index; LVEF, left ventricular ejection fraction; CI, confidence interval.

Follow-up echocardiography was unavailable in 22 patients. Compared with patients with available follow-up imaging, those without follow-up echocardiography had a lower BMI and a different distribution of NYHA functional class, whereas no significant differences were observed in age, sex, etiology, baseline LVEF, QRS duration, LBBB status, NT-proBNP, baseline CALLY index, or creatinine.

### 3.3 Association of the CALLY index with clinical outcomes

During a median follow-up of 18 months (IQR, 7–24 months), 56 of the 213 patients (26.3%) experienced MACE. MACE events decreased across increasing CALLY tertiles, with 35, 15, and 6 events occurring in the lowest, middle, and highest tertiles, respectively. Kaplan–Meier analysis demonstrated a significant difference in MACE-free survival across the three CALLY tertiles (log-rank *p* < 0.0001) (Figure 3). Patients in the highest tertile had the most favorable prognosis, whereas those in the lowest tertile had the highest risk of adverse cardiovascular events.

**Figure 3:**
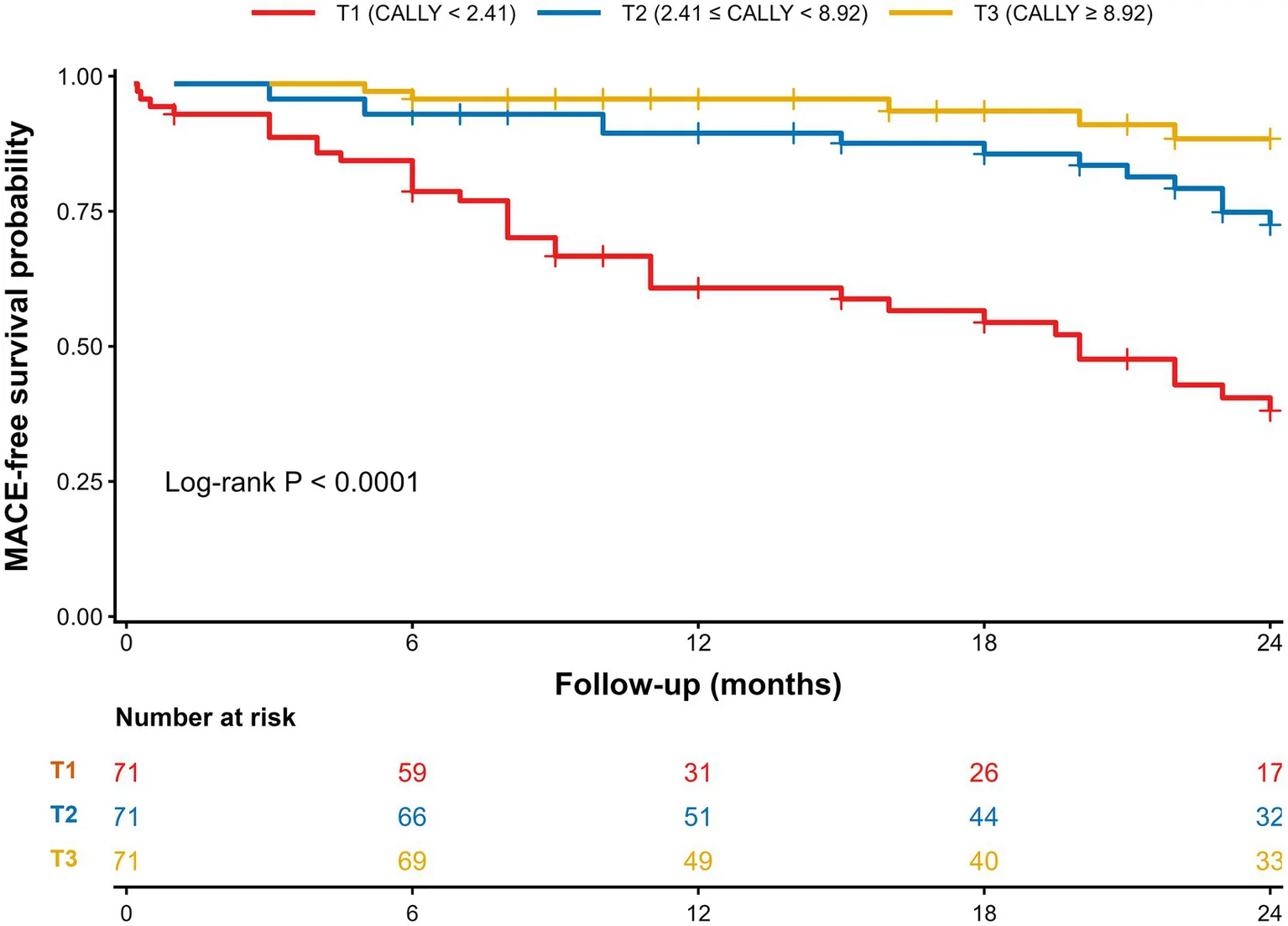
Kaplan–Meier analysis of MACE-free survival according to baseline CALLY index tertiles. The analysis included all 213 patients. CALLY tertile cutoffs (2.41 and 8.92) were derived from the baseline CALLY distribution in the full cohort, with 71 patients in each tertile. MACE-free survival differed significantly across CALLY tertiles (log-rank *p* < 0.0001). CALLY, C-reactive protein–albumin–lymphocyte index; MACE, major adverse cardiovascular events.

Among the 56 MACE events, 48 were heart-failure rehospitalizations and 8 were deaths; no heart transplantation or left ventricular assist device implantation occurred during follow-up. Across increasing CALLY tertiles, the numbers of heart-failure rehospitalizations were 30, 12, and 6, respectively, while the corresponding numbers of deaths were 5, 3, and 0.

Because the composite endpoint was predominantly driven by heart-failure rehospitalization, a Fine–Gray competing-risk analysis was performed, treating death as a competing event. After adjustment for age, sex, ischemic etiology, left bundle branch block, baseline left ventricular ejection fraction, and log2-transformed NT-proBNP, each doubling of the baseline CALLY index was associated with a 24.3% lower subdistribution hazard of first heart-failure rehospitalization [subdistribution hazard ratio (sHR), 0.757; 95% CI, 0.651–0.880; *p* < 0.001].

### 3.4 Independent association between the CALLY index and MACE and model performance

The association between the baseline CALLY index and MACE was evaluated using Cox proportional hazards regression models, with the CALLY index analyzed as a log2-transformed continuous variable. Hazard ratios (HRs) therefore represent the change in the hazard of MACE associated with each doubling of the CALLY index.

In the unadjusted model, each doubling of the CALLY index was associated with a lower hazard of MACE (HR, 0.746; 95% CI, 0.671–0.828; *p* < 0.001). The association remained essentially unchanged after adjustment for age and sex (HR, 0.753; 95% CI, 0.676–0.839; *p* < 0.001). In the principal multivariable model, after further adjustment for ischemic versus nonischemic etiology, left bundle branch block, baseline left ventricular ejection fraction, and log2-transformed NT-proBNP, each doubling of the baseline CALLY index was associated with a 24.9% lower hazard of MACE (HR, 0.751; 95% CI, 0.664–0.850; *p* < 0.001) (Table 3).

**Table 3:** Association between the baseline CALLY index and major adverse cardiovascular events in Cox proportional hazards regression models.

Model	HR per doubling of CALLY	95% CI	*P*-value
Unadjusted	0.746	0.671–0.828	<0.001
Model 1	0.753	0.676–0.839	<0.001
Model 2	0.751	0.664–0.850	<0.001

CALLY was analyzed as a log2-transformed continuous variable; therefore, hazard ratios represent the relative change in the hazard of MACE associated with each doubling of the CALLY index. Model 1 was adjusted for age and sex. Model 2 was additionally adjusted for ischemic versus nonischemic etiology, left bundle branch block, baseline left ventricular ejection fraction, and log2-transformed N-terminal pro-B-type natriuretic peptide. The proportional hazards assumption was satisfied for Model 2 (global *p* = 0.528). Bootstrap internal validation with 1,000 resamples yielded an apparent Harrell C-index of 0.738 and an optimism-corrected C-index of 0.705. HR, hazard ratio; CI, confidence interval; CALLY, C-reactive protein–albumin–lymphocyte index; MACE, major adverse cardiovascular events.

The proportional hazards assumption was satisfied for the principal model, with no significant violation for the CALLY index (*p* = 0.188) or for the model globally (*p* = 0.528).

To evaluate the incremental prognostic information provided by CALLY, a clinical model including age, sex, ischemic versus nonischemic etiology, LBBB status, baseline LVEF, and log2-transformed NT-proBNP was compared with the same model with the addition of log2-transformed CALLY. The Harrell C-index increased from 0.672 for the clinical model to 0.738 after addition of CALLY (ΔC-index = 0.066; 95% bootstrap CI, 0.015–0.138). The addition of CALLY also significantly improved model fit according to the likelihood-ratio test (χ^2^ = 19.164; *p* < 0.001), indicating incremental prognostic information beyond the parsimonious clinical model.

Bootstrap internal validation of the principal model including CALLY, using 1,000 resamples, yielded an apparent Harrell C-index of 0.738. The estimated optimism was 0.033, resulting in an optimism-corrected Harrell C-index of 0.705. The optimism-corrected C-index indicated moderate discrimination.

### 3.5 Time-dependent discrimination of the CALLY index

Time-dependent receiver operating characteristic (ROC) analyses were performed at 12 and 18 months to compare the discrimination of the CALLY index with the IBI, PLR, GNRI, and PNI for MACE.

The CALLY index showed the highest time-dependent area under the curve (AUC) among the five biomarkers at both 12 and 18 months (Table 4 and Figure 4). After Holm correction for multiple comparisons, the discrimination of CALLY was significantly greater than that of GNRI and PLR at both time points and greater than that of PNI at 12 months. The difference between CALLY and PNI was not statistically significant at 18 months. Although the AUCs for CALLY were numerically higher than those for IBI at both time points, these differences did not reach statistical significance after correction for multiple comparisons.

**Table 4:** Time-dependent discrimination of the CALLY index and comparator biomarkers for MACE at 12 and 18 months.

Biomarker	12-month AUC (95% CI)	Adjusted P vs. CALLY	18-month AUC (95% CI)	Adjusted P vs. CALLY
CALLY	0.773 (0.689–0.856)	Reference	0.759 (0.672–0.846)	Reference
IBI	0.736 (0.646–0.825)	0.089	0.726 (0.636–0.817)	0.094
PNI	0.655 (0.551–0.760)	0.037	0.655 (0.558–0.752)	0.067
GNRI	0.624 (0.515–0.733)	0.037	0.600 (0.497–0.702)	0.014
PLR	0.588 (0.475–0.700)	<0.001	0.555 (0.448–0.663)	<0.001

AUC, area under the curve; CALLY, C-reactive protein–albumin–lymphocyte index; IBI, inflammatory burden index; PLR, platelet-to-lymphocyte ratio; GNRI, geriatric nutritional risk index; PNI, prognostic nutritional index; MACE, major adverse cardiovascular events. Adjusted *P-*values represent pairwise comparisons with CALLY after Holm correction for multiple testing. CI, confidence interval.

**Figure 4:**
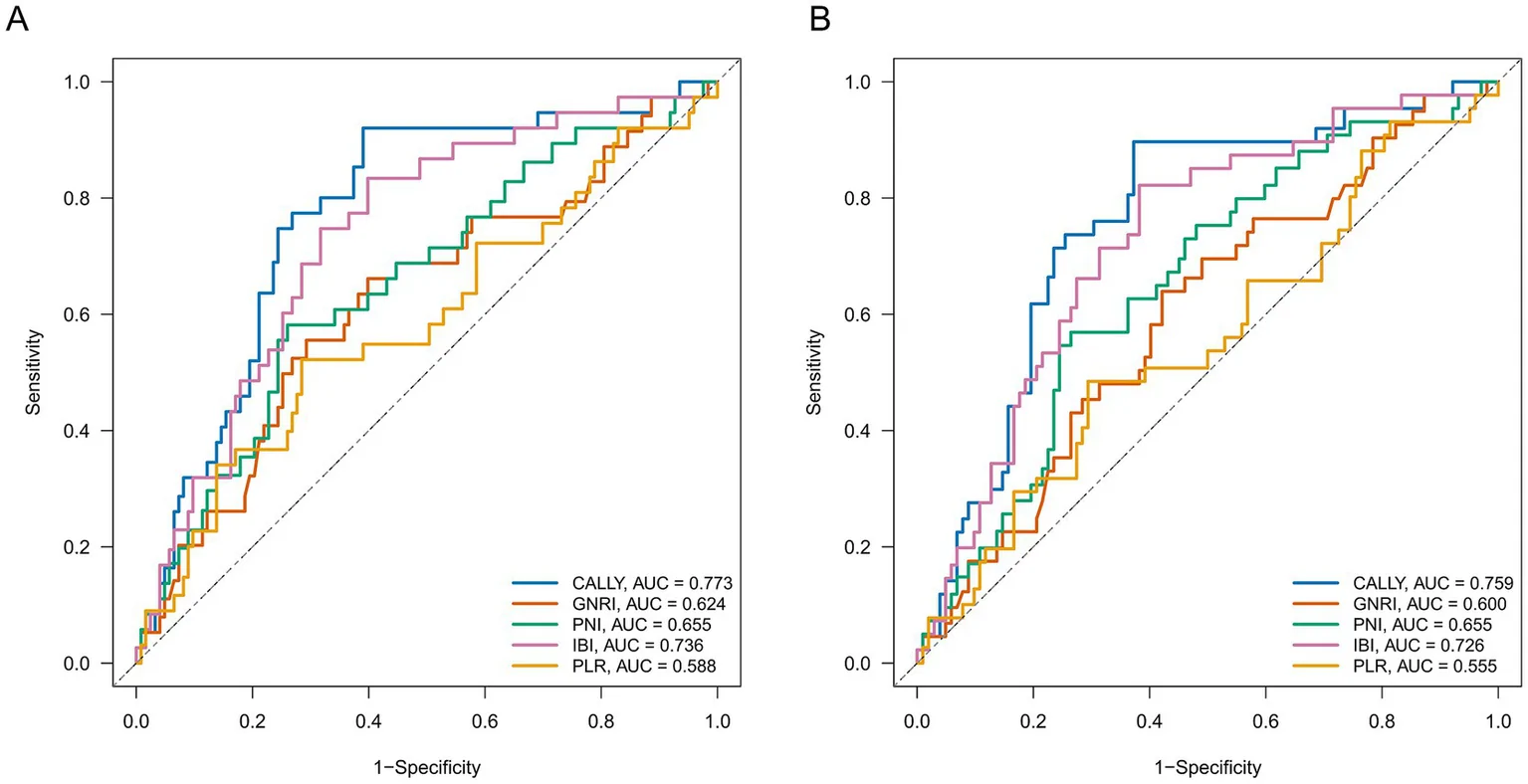
Time-dependent receiver operating characteristic (ROC) curves for MACE at 12 and 18 months. **(A)** Time-dependent ROC curves at 12 months. **(B)** Time-dependent ROC curves at 18 months. The discrimination of the CALLY index was compared with GNRI, PNI, IBI, and PLR. CALLY, C-reactive protein–albumin–lymphocyte index; GNRI, geriatric nutritional risk index; PNI, prognostic nutritional index; IBI, inflammatory burden index; PLR, platelet-to-lymphocyte ratio; MACE, major adverse cardiovascular events; AUC, area under the curve.

### 3.6 Nonlinear association between the CALLY index and MACE

Restricted cubic spline analysis was performed to further evaluate the association between baseline CALLY and MACE. CALLY was modeled on the log2 scale with adjustment for age, sex, ischemic versus nonischemic etiology, left bundle branch block, baseline LVEF, and log2-transformed NT-proBNP. A significant overall association was observed between the CALLY index and MACE (P for overall association <0.001), with evidence of nonlinearity (P for nonlinearity = 0.002) (Figure 5). The estimated hazard of MACE generally decreased with increasing CALLY levels, but the association was nonlinear. Confidence intervals were wider at the lower end of the CALLY distribution, indicating greater uncertainty in this range.

**Figure 5:**
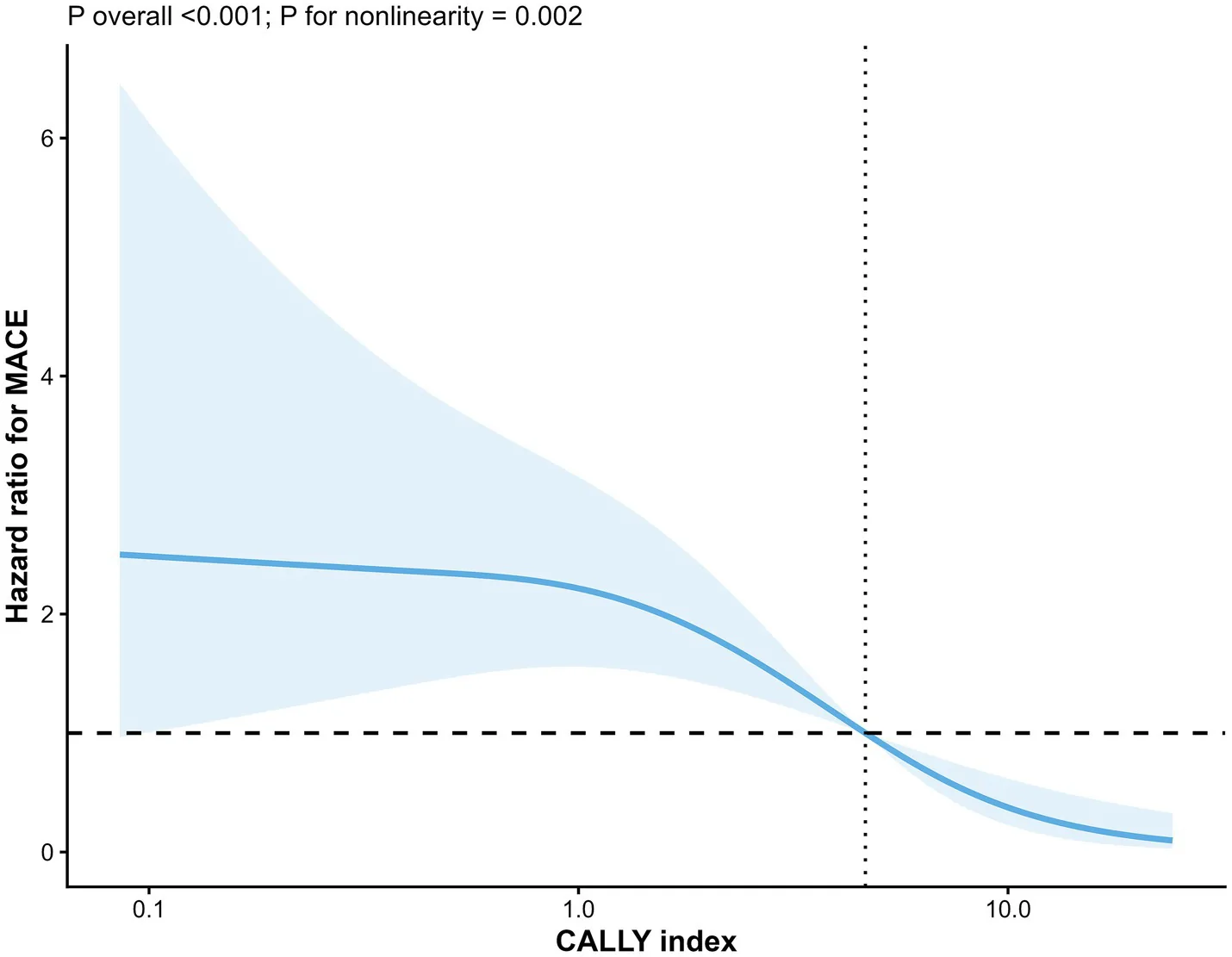
Restricted cubic spline analysis of the association between baseline CALLY index and MACE. The model was adjusted for age, sex, ischemic versus nonischemic etiology, left bundle branch block, baseline LVEF, and log2-transformed NT-proBNP. The solid line and shaded area show the estimated HR and 95% CI, respectively; the horizontal dashed line denotes HR = 1 and the vertical dotted line denotes the reference CALLY value. CALLY, C-reactive protein–albumin–lymphocyte index; MACE, major adverse cardiovascular events; LVEF, left ventricular ejection fraction; HR, hazard ratio; CI, confidence interval; NT-proBNP, N-terminal pro-B-type natriuretic peptide.

### 3.7 Subgroup analyses

Exploratory subgroup analyses were performed according to age, sex, body mass index (BMI), hypertension, diabetes mellitus, coronary heart disease, and documented arrhythmia status (Figure 6). Overall, the inverse association between the CALLY index and MACE was generally consistent across clinically relevant subgroups. No significant interactions were observed for age, sex, hypertension, diabetes mellitus, coronary heart disease, or arrhythmia status (all P for interaction >0.05).

**Figure 6:**
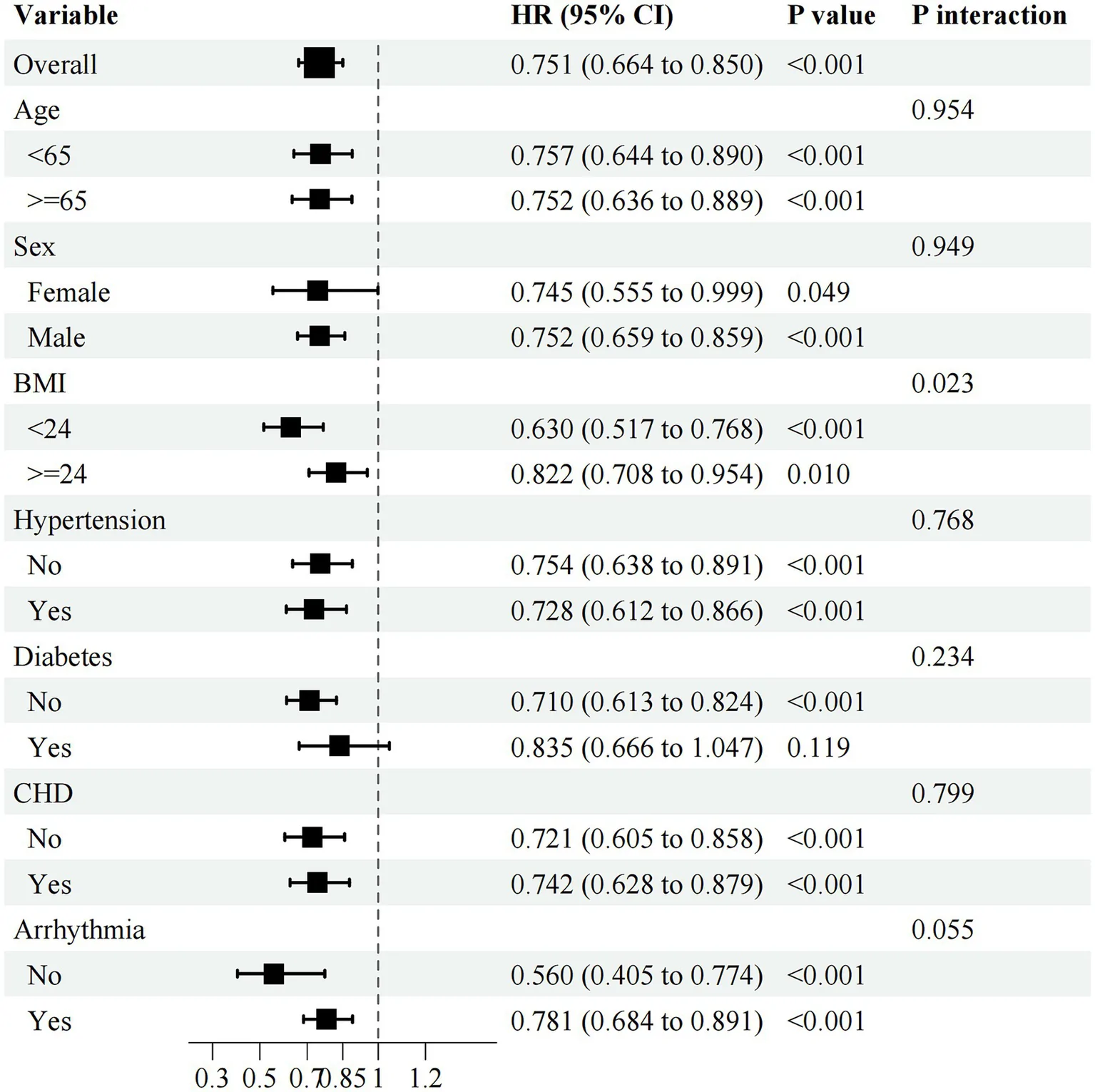
Exploratory subgroup analyses of the association between baseline CALLY index and MACE. Hazard ratios are reported per doubling of the CALLY index. *p-*values for interaction were used to assess potential effect modification across subgroups. CALLY, C-reactive protein–albumin–lymphocyte index; MACE, major adverse cardiovascular events; HR, hazard ratio; CI, confidence interval; BMI, body mass index; CHD, coronary heart disease.

A significant interaction was observed for BMI (P for interaction = 0.023). Each doubling of the CALLY index was associated with a lower hazard of MACE in both BMI strata, although the association was stronger among patients with BMI < 24 kg/m^2^ (HR, 0.630; 95% CI, 0.517–0.768; *p* < 0.001) than among those with BMI ≥ 24 kg/m^2^ (HR, 0.822; 95% CI, 0.708–0.954; *p* = 0.010). Given the exploratory nature of the subgroup analyses and the limited number of events within individual subgroups, this potential effect modification should be interpreted with caution.

### 3.8 Sensitivity analyses

Sensitivity analyses addressing heterogeneity in CRT indications yielded results consistent with the principal analyses. Restricting the cohort to patients with baseline LVEF ≤35% or excluding the 14 pacemaker-to-CRT upgrade patients did not materially change the associations of CALLY with MACE or LVEF improvement. In analyses stratified by baseline LVEF, higher CALLY was associated with a lower hazard of MACE in both LVEF groups, with no significant interaction (P for interaction = 0.795). No significant interaction was observed for LVEF improvement (P for interaction = 0.284) (Supplementary Table S1).

Calendar-era analyses also showed consistent results. Baseline CALLY levels did not differ significantly between 2015–2020 and 2021–2025, whereas SGLT2 inhibitor use was higher in the later period. Additional adjustment for calendar era, implantation year, or SGLT2 inhibitor use did not materially change the association between CALLY and MACE. No significant interaction by calendar era was observed for either MACE (P for interaction = 0.763) or LVEF improvement (P for interaction = 0.821) (Supplementary Table S1).

CR*p* values at or below the lower detection limit were present in 49 patients (23.0%). Sensitivity analyses using LOD/√2, LOD/2, and left-censored lognormal multiple imputation yielded similar associations with MACE (HRs, 0.755–0.766; all *p* < 0.001) and LVEF improvement (β, 0.733–0.879; all *p* ≤ 0.008) (Supplementary Table S2).

## 4 Discussion

In this multicenter retrospective cohort study, the baseline CALLY index was independently associated with LVEF improvement and subsequent clinical outcomes in patients undergoing CRT. Taken together, these findings suggest that the inflammation–nutrition–immune status reflected by the CALLY index may provide complementary information beyond conventional electrical and echocardiographic characteristics.

Chronic inflammation, malnutrition, and immune dysfunction are increasingly recognized as interrelated mechanisms driving the progression of heart failure (8–11). Persistent inflammatory activation promotes myocardial fibrosis, adverse ventricular remodeling, oxidative stress, and neurohormonal activation, whereas impaired nutritional status reduces metabolic reserve and limits tissue repair. At the same time, immune dysfunction contributes to persistent inflammation and progressive cardiac dysfunction (20–23). Because these biological processes interact throughout the course of HF, assessment of a single pathway may not adequately reflect the overall physiological condition of patients. The association between CALLY and LVEF improvement supports the concept that a more favorable inflammation–nutrition–immune profile may be associated with greater LVEF improvement after CRT. Nevertheless, because volumetric measures such as left ventricular end-systolic volume (LVESV) were not available, the echocardiographic findings should be interpreted specifically in terms of LVEF improvement rather than direct evidence of ventricular reverse remodeling. In addition, CRT-related remodeling may involve both ventricles, and improvement in right-ventricular function after CRT has also been reported (24); these changes could not be evaluated in the present study.

The CALLY index integrates three routinely available laboratory parameters—C-reactive protein, serum albumin, and lymphocyte count—into a single composite biomarker reflecting inflammatory burden, nutritional status, and immune competence. Compared with isolated inflammatory or nutritional markers, this multidimensional assessment may better characterize the overall biological condition of patients before CRT implantation. In the present study, CALLY showed favorable discrimination compared with several commonly used inflammatory and nutritional indices, although its performance was not consistently superior, particularly to IBI. In addition, the nested model comparison suggested that CALLY provided incremental prognostic information beyond the parsimonious clinical model. Nevertheless, the optimism-corrected C-index indicated only moderate overall discrimination. Accordingly, CALLY should be viewed as a complementary marker rather than a stand-alone prediction tool.

Previous studies have demonstrated that systemic inflammation and poor nutritional status are associated with adverse ventricular remodeling, heart failure hospitalization, and reduced survival in patients with HF (8–11, 25–29). More recently, the CALLY index has shown prognostic value in HF and several other cardiovascular diseases (12–15). A recent large cohort of patients with chronic heart failure further demonstrated an independent association between higher CALLY levels and more favorable cardiovascular outcomes (17). Nutritional assessment has also been investigated specifically in CRT populations; notably, GNRI has been associated with treatment response and long-term prognosis after CRT (18). Our findings extend this evidence to patients undergoing CRT by evaluating the association of CALLY with both LVEF-based functional improvement and clinical outcomes. Thus, the novelty of the present study lies primarily in the CRT-specific application of CALLY rather than in establishing CALLY as a new prognostic biomarker for HF in general. CRT practice has also evolved during the study period, with conduction-system pacing increasingly considered as an alternative to conventional biventricular CRT. Recent consensus recommendations and a meta-analysis of randomized trials have further highlighted this evolution in contemporary CRT practice (30, 31).

The composition of the clinical endpoint also warrants consideration. Because the composite outcome was predominantly driven by heart-failure rehospitalization and relatively few deaths occurred, the present findings mainly reflect heart-failure morbidity rather than mortality. The competing-risk analysis, in which death was treated as a competing event, provided additional support for the association with first heart-failure rehospitalization. Therefore, these findings should not be interpreted as evidence of a mortality or survival benefit.

The spline analysis suggested a nonlinear association between CALLY and clinical outcomes. However, uncertainty was greater at the extremes of the CALLY distribution, and the present data do not support a specific clinical threshold. Similarly, the stronger association observed among patients with lower BMI should be considered exploratory. Given the limited number of events within individual subgroups and the multiple comparisons involved, this potential effect modification requires confirmation in larger independent cohorts.

The present study has several potential clinical implications. First, the CALLY index is inexpensive, readily available, and can be calculated from routine laboratory tests without additional cost or specialized equipment, supporting its further evaluation as an adjunctive biomarker in patients undergoing CRT. Second, the biological information provided by the CALLY index may complement rather than replace established electrocardiographic and echocardiographic parameters in preprocedural assessment. Finally, patients with a less favorable inflammation–nutrition–immune profile may represent a clinically vulnerable subgroup that warrants further prospective evaluation. However, the present observational data do not support using CALLY to determine CRT candidacy or to guide treatment decisions. Prospective external validation is required before its incremental clinical utility can be established. Whether modification of inflammatory or nutritional status can improve LVEF response or clinical outcomes after CRT remains to be determined.

## 5 Study limitations

This study has several limitations. First, its retrospective observational design precludes causal inference, and residual confounding cannot be excluded. Second, the sample size and number of clinical events were relatively modest, and external validation in larger independent cohorts is required. Third, follow-up echocardiography was unavailable in some patients and was based mainly on changes in LVEF rather than volumetric measures, which may have introduced selection bias and limited assessment of structural ventricular remodeling. Fourth, although sensitivity analyses according to pacemaker upgrade status and calendar era yielded consistent findings, residual confounding cannot be excluded because detailed information on LV lead position, effective biventricular pacing, AV-junction ablation, and contemporary HF therapy was not uniformly available. Finally, the CALLY index was assessed only at baseline; therefore, the prognostic significance of longitudinal changes could not be evaluated.

## 6 Conclusion

In this multicenter retrospective cohort study, a higher baseline CALLY index was independently associated with greater LVEF improvement and a lower hazard of MACE after CRT. The association with MACE predominantly reflected heart-failure rehospitalization. CALLY provided incremental prognostic information beyond a parsimonious clinical model, although overall discrimination after optimism correction was moderate. These findings support further prospective external validation of CALLY as a complementary biomarker rather than its use for clinical decision-making.

## Data Availability

The raw data supporting the conclusions of this article will be made available by the authors, without undue reservation.
