# Supplementary material for: The C-reactive protein–albumin–lymphocyte index is associated with ventricular functional improvement and long-term outcomes after cardiac resynchronization therapy
Source: Front Nutr. 2026 Sep 18;13:1961200. doi: 10.3389/fnut.2026.1961200 (PMC13630771; doi:10.3389/fnut.2026.1961200)
Supplement: Supplementary file 1 [file Table_1.docx]

**Supplementary Table 1.** Sensitivity and stratified analyses of the associations of baseline CALLY index with MACE and LVEF improvement according to baseline LVEF, pacemaker upgrade status, and calendar era.

| **Analysis** | **N** | **MACE events** | **HR per doubling of CALLY (95% CI)** | **P value** | **N for LVEF analysis** | **β for ΔLVEF per doubling of CALLY, percentage points (95% CI)** | **P value** |
| --- | --- | --- | --- | --- | --- | --- | --- |
| **A. Sensitivity analyses according to CRT population** |  |  |  |  |  |  |  |
| Baseline LVEF ≤35% | 146 | 41 | 0.740 (0.634–0.864) | <0.001 | 130 | 1.145 (0.360–1.931) | 0.005 |
| Excluding pacemaker-to-CRT upgrades | 199 | 55 | 0.755 (0.667–0.855) | <0.001 | 181 | 0.985 (0.333–1.637) | 0.003 |
| **B. Stratified analyses by baseline LVEF** |  |  |  |  |  |  |  |
| LVEF ≤35% | 146 | 41 | 0.740 (0.634–0.864) | <0.001 | 130 | 1.145 (0.360–1.931) | 0.005 |
| LVEF >35% | 67 | 15 | 0.681 (0.518–0.896) | 0.006 | 61 | 0.330 (−0.802 to 1.462) | 0.570 |
| **P for interaction** | - | - | - | **0.795** | - | - | **0.284** |
| **C. Calendar-era sensitivity analyses** |  |  |  |  |  |  |  |
| **Additional adjustment for calendar era** | 213 | 56 | **0.752 (0.663–0.852)** | <0.001 | 191 | 0.930 (0.286–1.575) | 0.005 |
| Additional adjustment for implantation year | 213 | 56 | 0.752 (0.664–0.851) | <0.001 | 191 | 0.945 (0.304–1.586) | 0.004 |
| Additional adjustment for SGLT2 inhibitor use | 213 | 56 | 0.749 (0.661–0.849) | <0.001 | - | **-** | - |
| **D. Stratified analyses by calendar era** |  |  |  |  |  |  |  |
| 2015–2020 | 77 | 28 | 0.728 (0.572–0.928) | 0.010 | 70 | 0.820 (−0.403 to 2.044) | 0.194 |
| 2021–2025 | 136 | 28 | 0.735 (0.619–0.873) | <0.001 | 121 | 0.951 (0.157–1.746) | 0.021 |
| **P for interaction** | - | - | - | **0.763** | - | **-** | **0.821** |

Abbreviations: CALLY, C-reactive protein–albumin–lymphocyte index; CI, confidence interval; CRT, cardiac resynchronization therapy; HR, hazard ratio; LBBB, left bundle branch block; LVEF, left ventricular ejection fraction; MACE, major adverse cardiovascular events; NT-proBNP, N-terminal pro-B-type natriuretic peptide; SGLT2, sodium–glucose cotransporter 2.

CALLY was analyzed as a log2-transformed continuous variable; therefore, HRs and β coefficients represent the association with each doubling of the baseline CALLY index. For MACE, Cox proportional hazards models were adjusted for age, sex, ischemic versus nonischemic etiology, LBBB status, baseline LVEF, and log2-transformed NT-proBNP. Calendar era, implantation year, or baseline SGLT2 inhibitor use was additionally included in the corresponding sensitivity model as indicated. For the echocardiographic analyses, absolute change in LVEF (follow-up LVEF minus baseline LVEF) was analyzed using multivariable linear regression adjusted for age, sex, etiology, LBBB status, baseline LVEF, and preprocedural QRS duration, with HC3 robust standard errors. Calendar era or implantation year was additionally included in the corresponding sensitivity model as indicated. Calendar era was categorized as 2015–2020 and 2021–2025. Interaction P values were derived from formal tests of CALLY-by-baseline-LVEF-category or CALLY-by-calendar-era interactions, as applicable.

**Supplementary Table 2.** Sensitivity analyses of the associations of the CALLY index with MACE and LVEF improvement using alternative approaches for CRP values at the lower limit of detection.

| **CRP handling method** | **N** | **MACE events** | **HR per doubling of CALLY (95% CI)** | **P value** | **N for LVEF analysis** | **β for ΔLVEF per doubling of CALLY, percentage points (95% CI)** | **P value** |
| --- | --- | --- | --- | --- | --- | --- | --- |
| Primary analysis: LOD (0.5 mg/L) | 213 | 56 | 0.751 (0.664–0.850) | <0.001 | 191 | 0.949 (0.313–1.586) | 0.004 |
| LOD/√2 | 213 | 56 | 0.755 (0.669–0.852) | <0.001 | 191 | 0.879 (0.284–1.473) | 0.004 |
| LOD/2 | 213 | 56 | 0.760 (0.675–0.855) | <0.001 | 191 | 0.804 (0.248–1.359) | 0.005 |
| Left-censored lognormal multiple imputation (100 imputations) | 213 | 56 | 0.766 (0.680–0.862) | <0.001 | 191 | 0.733 (0.193–1.272) | 0.008 |

Abbreviations: CALLY, C-reactive protein–albumin–lymphocyte index; CI, confidence interval; CRP, C-reactive protein; HR, hazard ratio; LBBB, left bundle branch block; LOD, lower limit of detection; LVEF, left ventricular ejection fraction; MACE, major adverse cardiovascular events; NT-proBNP, N-terminal pro-B-type natriuretic peptide.

The lower limit of detection for CRP was 0.5 mg/L. In the primary analysis, CRP values recorded at or below the lower detection limit were assigned 0.5 mg/L. Sensitivity analyses replaced these values with LOD/√2 or LOD/2, respectively, or used stochastic multiple imputation from a fitted left-censored lognormal CRP distribution with 100 imputations. CALLY was recalculated under each approach and analyzed as a log2-transformed continuous variable; therefore, HRs and β coefficients represent the association with each doubling of CALLY. Cox models for MACE were adjusted for age, sex, ischemic versus nonischemic etiology, LBBB status, baseline LVEF, and log2-transformed NT-proBNP. Linear regression models for absolute change in LVEF were adjusted for age, sex, etiology, LBBB status, baseline LVEF, and preprocedural QRS duration, with HC3 robust standard errors.
